# Efficacy of p62-expressing plasmid in treatment of canine osteoarthritis

**DOI:** 10.21203/rs.3.rs-5461004/v1

**Published:** 2024-11-21

**Authors:** Vladimir Gabai, Evgeny Bakin, Maxim Harold Langs, Robert Delvin, Sergei Krasny, Yauheni Baranau, Sergey Polyakov, Maksim Patapovich, Sergey Gvozdev, Maksim Kardash, Aliaksei Bazyleuski, Andrei Yeliseyeu, Egor Lelikov, Andrei Barodka, Alexander Shneider

**Affiliations:** CureLab Vet; Institute of Bioinformatics Research and Education (IBRE), Belgrade, Serbia.; Miramonte High School, Orinda, CA 94563; Curelab Vet; N.N. Alexandrov National Cancer Centre of Belarus Minsk, Belarus; Minsk City Clinical Oncologic Centre; N.N. Alexandrov National Cancer Centre of Belarus Minsk; Faculty of Biology, Belarusian State University; BELVITUNIFARM, Belarus; Veterinary clinic Doctor Vet. Minsk; Dr.A.Bazylevsky Veterinary Center, Belarus; Veterinary clinic AZBUKAVET, Grodno; PoliVetClinic Gomel; Alpha-Vet Belarus; CureLab

**Keywords:** DNA vaccine, inflammation, chronic pain, clinical trial, safety

## Abstract

**Introduction:**

Osteoarthritis (OA) is a progressive degenerative disease of synovial joints which is highly prevalent in dogs and results in lameness, loss of joint function and mobility, chronic pain, and reduced quality of life. Traditional OA management consist of non-steroidal anti-inflammatory drugs and remains challenging because of significant side effects, thus there is an urgent need for new effective and safe therapeutics for OA.

**Methods:**

Here we present the results of our one-arm open-label pilot clinical study of our novel biologics, a DNA plasmid encoding SQSTM/p62, in 17 companion dogs suffering from OA. The dogs were injected intramuscular with p62-plasmid once a week for 10 weeks, and pain relief was measured using the CBPI (canine brief pain inventory) validated scale. Assessment by the owners was done weekly. The 11 parameters of CBPI are grouped in three major domains: pain severity score (PSS), pain interference score (PIS) and overall impression of the quality of life (QoL).

**Results:**

Treatment with the p62-plasmid improved all 11 parameters of CBPI as well as PSS, PIS and QoL: mean PSS score after the treatment decreased from 5.25 to 3.25, PIS score - from 7.0 to 3.27, and number of dogs with excellent and good QoL due to treatment increased from 1 to 12. Overall, the treatment success rate (i.e. a reduction ≥1 in PSS and ≥ 2 in PIS) was 90%. Importantly, similar to our previous studies with dogs and humans, no significant side effects of the p62-plasmid during the whole treatment period were observed.

**Discussion:**

We believe that anti-inflammatory effects of the p62-plasmid, which we described in our previous works, may play an important role in observed clinical benefits and it is worthy of further studies as a novel OA treatment modality.

## Introduction

Osteoarthritis (OA) is a progressive degenerative disease of synovial joints that characterised by structural and functional changes to the cartilage due to biomechanical and metabolic alterations. In humans, OA is the most common cause of disability worldwide among the elderly. OA is also highly prevalent in dogs with about 20% of the canine population older than 1 year is affected by the disease (1). This results in lameness, loss of joint function and mobility, chronic pain, and reduced quality of life (2).

Chronic states of inflammation (e.g., metabolic syndrome or aging), where synovitis is often present in the knees of patients with OA, appear important contributor to disease pathogenesis. Pro-inflammatory cytokines such as IL-1 and TNF are central to joint degeneration of OA, as well as to sensitization of pain neurons that innervate the joint capsule (3). Produced by chondrocytes and synoviocytes (fibroblasts and macrophages), these cytokines promote the cartilage-damaging activities of these cells (4) via, in particular, induction of metalloproteases (5). Pro-inflammatory cytokines, therefore, may be important targets for pharmaceutical intervention. Other than surgical management for a select group of arthritic dogs, there are no disease-modifying therapies with strong evidence of efficacy in canine OA. Therefore, its management is mostly based on relieving the symptoms of the disease by treating pain and inflammation, improving mobility and hence quality of life, whilst protecting joints from OA.

Non-steroidal anti-inflammatory drugs (NSAIDs) are still considered the cornerstone for the management of canine OA. However, in many cases, pain reduction is inadequate (6) and NSAID have deleterious effects when prescribed over long durations (7). They, in particular, can increase blood pressure, cause gastric ulcers, and even sometimes lead to acute kidney failure, stroke, or myocardial infarction. Chronic systemic use of another common treatment modality, steroids, can lead to osteoporosis, osteonecrosis, Cushing’s disease, adrenal insufficiency, hyperlipidemia, and other adverse effects (8). Thus, OA-related pain management remains challenging and there is an urgent need for new effective and safe therapeutics for OA.

We have recently developed a biologic double-stranded supercoiled circular plasmid DNA coding for the protein SQSTM1/p62 (Elenagen). This protein plays multiple functions in the cells, controlling autophagy, signal transduction, inflammation and others (9). Since p62 is dispensable for normal cells, but it is essential for tumor cells, it can be a good target for anticancer vaccine (10). Indeed, Elenagen revealed anti-cancer activity in canine mammary tumors (11) and in clinical trials in human ovary cancer plus, demonstrated a good safety profile (12, 13) (14). During our studies in animals, we found a quite unexpected effect of Elenagen – it alleviated diseases of chronic inflammation (see ref (9) for review. We found that Elenagen decreases generation of pro-inflammatory cytokines such as TNF, IL-1, IL-6, and increases anti-inflammatory cytokines (e.g. IL-4 and IL-10) in a several animal disease models (9). Such unique multi-modal effect of Elenagen, as we found, is conducive for alleviation of symptoms in diseases of chronic inflammation. Among them are osteoporosis (15), metabolic syndrome/obesity (16), age-related macular degeneration (AMD) (17) and Alzheimer disease (18). Given that OA is also considered as disease of chronic inflammation, and canine OA is much closer to human OA than rodent models, we conducted this pilot study of the p62-plasmid in companion dogs.

## Materials and Methods

This one-arm open label study of 17 companion dogs with OA was conducted in veterinary clinics in the Republic of Belarus in 2023–2024. All owners provided written confirmation of informed consent.

### Animal selection

Client-owned dogs weighing ≥ 3 kg of any age, sex and breed with a medical history, clinical signs, physical examination findings, and radiographic findings consistent with OA were recruited for inclusion in the study. Radiographic finding by radiologists included subchondral bone sclerosis, bone remodelling, osteophytes, irregular or diminished joint space at least at one joint. Only dogs with a diagnosis of OA made while screening for the study that had not commenced treatment of any type (including nutraceuticals, special diets, and over-the-counter supplement-type products) and dogs with a prior diagnosis of OA that the owners had elected not to treat were eligible for inclusion in the study. Animals were confirmed to be in good general health based on a general physical examination and routine blood (hematology and biochemistry) tests.

Dogs are excluded from the study if received:

1. NSAIDs during the 2 weeks prior to evaluation for study enrolment,
2. glucocorticoids during the 4 weeks prior to evaluation, or
3. opioids during the 4 weeks prior to evaluation.

Also excluded:

1. dogs with any clinically important neurologic disease or orthopedic disease other than OA, as determined on the basis of history and results of a physical examination,
2. dogs with any chronic disease that required daily medication,
3. dogs with a history of coagulopathy and unexplained bleeding episodes;
4. dogs with clinically important abnormalities detected on a complete blood count and serum biochemical testing at the time of the initial evaluation.

### Procedures and Outcome measurement

The trial is a single-arm prospective interventional trial. The dogs are treated with the p62-plasmid, 1 mg intramuscular (IM) once a week for 10 weeks, and the efficacy was assessed by the owners every week using the Canine Brief Pain Inventory (CBPI). CBPI consists of three domains: the pain severity score (PSS, 0–10 scale), the pain interference score (PIS, 0–10) and the overall impression by an owner of the quality of life (QoL, “poor”, “fair”, “good”, “excellent”). To be eligible for the study, initial PSS > 2 and/or PIS > 2 were required.

The primary efficacy endpoint was treatment success at 10 weeks based on owner assessment of pain using CBPI. Treatment success was defined as a reduction ≥ 1 in PSS (0–10; 0 no pain, 10 extreme pain) and ≥ 2 in PIS (0–10; 0 no pain, 10 extreme pain) following the CBPI author recommendation (19, 20) compared with pre-treatment (baseline). Secondary efficacy endpoints included CBPI-based treatment success for all other assessed time points, the owner assessed PSS and PIS scores, and QoL and the percentage of dogs classified as having a good’ or ‘excellent’ QoL at a final time point.

### Statistical methods

Quantitative data were described with medians and quartiles. Pre- and Post analysis of PSS, PIS and QoL was performed with a paired Mann-Whitney test. Additionally in each subject and each score we quantified a trend of improvements via Spearman correlation *ρ* between the score value and time after the first intervention. Thus, in case of monotonic improvement/deterioration we would expect to see a *ρ* of −1 or + 1 respectively. Afterward, a two-sided t-test was used to test the hypothesis that the expectation of *ρ* is 0. A threshold of 0.05 was chosen as a significance level, all tests were two-sided. Bonferroni-Holm adjustment was used for multiple testing corrections.

## Results

### Patient’s characteristics

Characteristics of the dogs enrolled in the study are presented in Table 1. As expected, most dogs with OA are Large breeds (e.g., 6 of 17 are Labradors), and of rather old age (median = 9 years), although smaller and younger dogs were also presented (e.g., 5-yr old Spitz, #9, Table 1). All dogs demonstrated chronic OA by radiographic finding and clinical signs, but were otherwise were in a good health.

### Efficacy of treatment of p62 olasmid in dogs with OA

**Figure 1:**
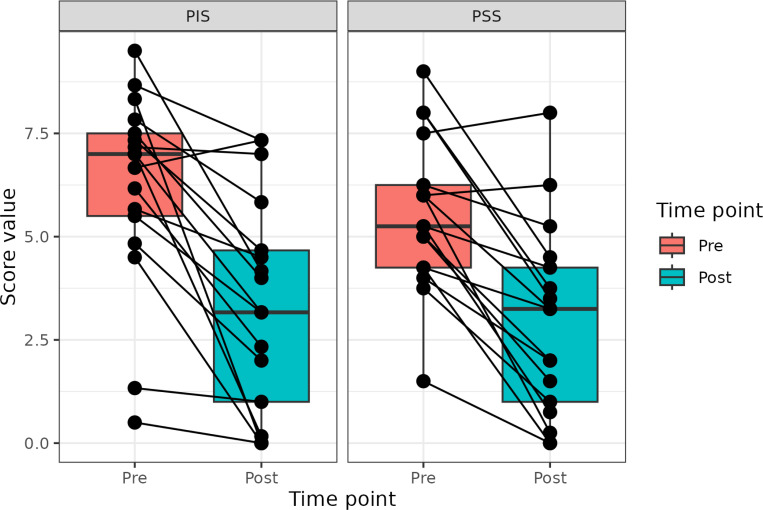
Changes in PIS and PSS scores after the treatment with p62 plasmid (described with a median, Q1 and Q3).

The dogs enrolled in the study were treated IM with 1 mg of the p62-plasmid once a week for 10 weeks. As in previous studies in dogs and humans, no adverse effects of the treatment were observed. For assessment of the treatment effect we used standard CBPI test which was done by dog owners every week. The changes in main components of CBPI, the pain severity score (PSS), pain interference score (PIS), and quality of life (QoL) in individual dogs after treatment are presented in Table 2 and Fig. 1

Based on CBPI assessment, only one dog (#13) demonstrated some worsening of CBPI scores, although less than 1 unit in value, and this dog was the oldest one in the population (14 year old Labrador retriever) (Table 1). Another dog, #9, show worsening of QoL (from fair to poor), and this was the smallest dog (Pomeranian Spitz). All other dogs demonstrated a various degree of improvement in all three component of CBPI score, with some of them (#3, #8) show disappearance of all symptoms of OA by the end of the treatment (Table 1). Thus, as whole, the treatment with the p62-plasmid was effective in 15 of 17 dogs (90%).

Change in PSS and PIS scores for all dogs is presented in Fig. 1. Both PSS and PIS scores are decreased significantly by the end of the treatment. Specifically, average PSS score decreased from 5.25 to 3.25 (p < 0.001) and average PIS score - from 7.0 to 3.17, (p < 0.001). Also a significant improvement in QoL was observed: while only 6% of dogs had good QoL before treatment, it increased to 53% after the treatment (Fig. 1)

Besides assessment by CBPI, we also made short video clips of dog’s behaviour before and after the study. These clips also demonstrate improvement in dog’s mobility after the treatment (see https://drive.google.com/drive/folders/1sBmjxvcC9lWOMLLaTPATTCi23SdtPynK)

**Figure 2:**
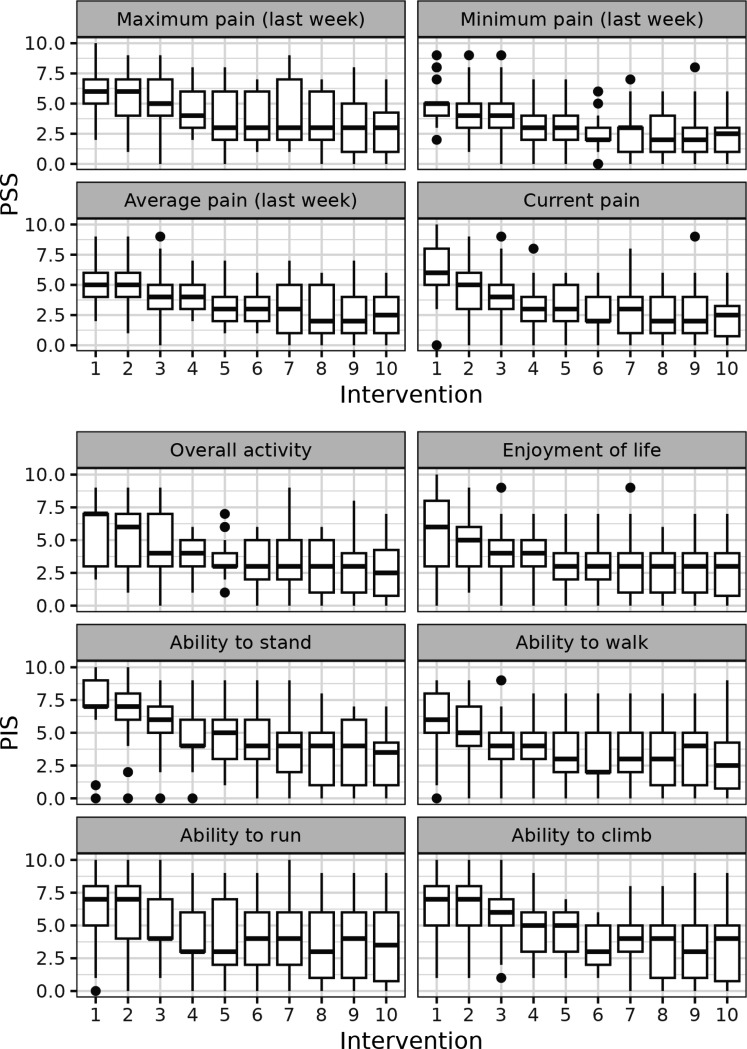
CBPI score dynamics during the treatment with p62 plasmid

We next addressed the question how each of 10 components of CBPI score changed before and during treatment in whole dog population. The positive effect of the treatment on all parameters started to observe mostly after 2–4 weeks of treatment, whereas after 5–6 weeks of the treatment the parameters reached the plateau, Fig. 2.

**Figure 3:**
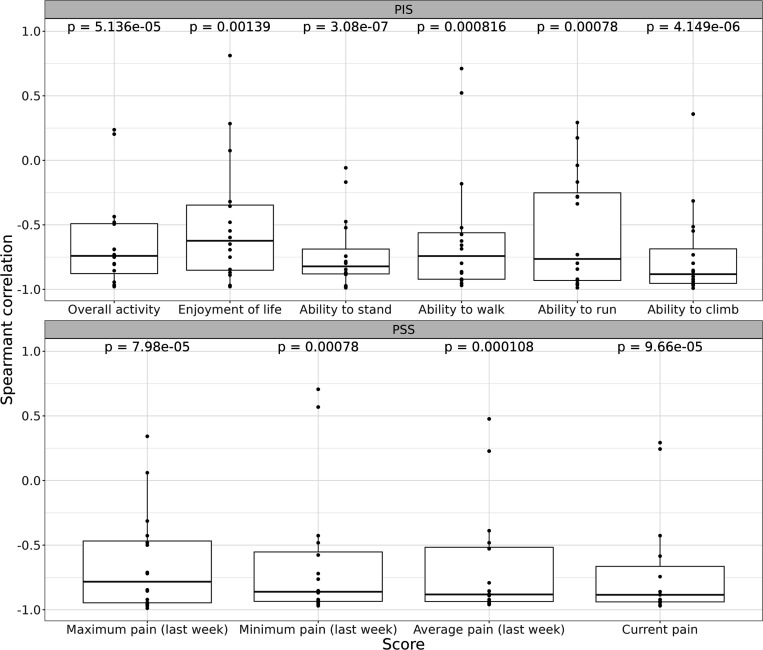
Spearman correlation *ρ* for each parameter of subjective assessment (CBPI score). See Materials and Methods for details.

To understand the relationship between the duration of injections time and the behavioral scores, we calculate the Spearman correlation coefficients. We graph the Spearman correlation coefficients for each subjective assessment in Fig. 3. The calculations show that for all 10 parameters of CBPI scores there is a clear inverse correlation between scores and the time of the treatment, with several parameters (e., pain levels) close to −1.0 (Fig. 3). Overall, correlation of all parameters with time of treatment demonstrates statistical significance ranging from 4×10^− 6^ to 0.0014 (Fig. 3).

## Discussion

OA is regarded as a significant cause of pain, lameness and morbidity in humans, dogs and some other animals (21) (22). It is the most common cause of disability among the elderly dogs, and the incidence of OA in younger individuals is also rising likely due to the increase in obesity and post-traumatic osteoarthritis (8). OA in dogs is associated with a variety of clinical signs such as stiffness, lameness, and gait alterations (23).

There is currently no cure for OA and most of the treatments are basically symptomatic in order to manage pain, stiffness and swelling (24). In other words, the goal of available therapies is to delay the progression of the disease, reduce pain and restore mobility, with the final objective to improve the overall quality of life (22, 25).

Here we present the results of our pilot clinical study of a novel biologic, a DNA plasmid encoding SQSTM/p62, in companion dogs suffering from OA. The dogs in the study were injected IM with the p62-plasmid once a week for 10 weeks. As the main criterion for clinical efficacy we employed CBPI that was assessed weekly by the owners. CBPI is widely used in OA studies in dogs and include 11 parameters constituting three major domains: the pain severity score (PSS) the pain interference score (PIS) and the overall impression of the quality of life (QoL) (19, 20)

We found that the treatment with the p62-plasmid significantly improved all 11 parameters of CBPI as well as PSS, PIS and QoL (Fig. 1, Table 2, 3). Specifically, mean PSS score after the treatment decreased from 5.25 to 3.25 (i.e. 1.6 times), PIS score - from 7.0 to 3.27 (2.2 times), and number of dogs with excellent and good QoL due to treatment increased from 1 to 12 (Table 2). Overall, the treatment success rate (i.e. a reduction ≥ 1 in PSS and ≥ 2 in PIS) was 90%. Importantly, similar to our previous studies with dogs and humans, no side effects of the p62-plasmid during the whole treatment period were observed.

Analysing the time course of changes in CBPI scores during the treatment, we observed that all parameters of CBPI significantly decreased with time although with somewhat different dynamics (Fig. 2, 3). The scores mostly started to improve after 3–4 injections (i.e. after 3–4 weeks), and reached the plateau after approximately 5–6 injections (Fig. 2, 3).

We compared the results of our study with previous trials of other anti-OA drugs in dogs. For instance, in dogs receiving NSAID drug carprofen, there was decrease in median PSS scores from 4.25 to 2.25, and PIS score from 4.33 to 2.67 on days 0 and 14, respectively (19). In the trial of currently approved anti-NGF monoclonal Ab (Librela, Zoetis), decrease in PSS score was from 4.1 to 3.0, and PIS score – from to 3.2 after 6 weeks of treatment with subsequent plateau till 12 weeks (end of the trial); the treatment success rate being 57% comparing to 34% in placebo group (26) https://www.librelavetteam.com/. Although the number of dogs in our pilot trial was small, all results are statistically significant and, comparing to other currently used medications mentioned above, efficacy of the p62-plasmid for treatment of OA looks very promising.

Chronic inflammation is currently recognized as an important contributor in pathogenesis of OA. The main pro-inflammatory cytokines thought to be involved in its pathogenesis are TNF-a and IL-1b, which act on synoviocytes and chondrocytes through their receptors (4). Accordingly, increased levels of IL-1b and TNF-a have been found in the synovial fluids of dogs with OA compared to unaffected joints (27).

Besides inflammatory cytokines, there are also various anti-inflammatory cytokines that play a positive role in OA; these include IL-4, IL-10, IL-13 and IL-1b receptor antagonist (IL-1Ra) (28). For instance, intra-articular injections of a plasmid expressing IL-10 reduced pain measures in the dogs, based on veterinary and owner ratings, without any adverse findings (29).

Our p62-expressing plasmid was originally developed as anticancer biological and it demonstrated broad-range anti-tumor activity in preclinical studies (30). Also, it showed efficacy in mammary tumors in dogs (11) and advanced solid tumors in humans (12, 13) (14). During these studies, we made an unexpected observation that the p62-plasmid can also affect bone development in mice. As we observed, the p62-plasmid administration could “instruct” mesenchymal stem cells (MSCs) to release growth and differentiation factors, as well as anti-inflammatory cytokines, that together exerted a bone-forming action (31) (32). Accordingly, p62 DNA-plasmid injection to ovariectomized (OVX) mice or rats reversed the osteopenic/inflammatory milieu and restored bone micro-architecture via new bone deposition (15, 32). In particular, the p62-plasmidinduced the transcription of main pre-osteoblast differentiation markers and alleviates inflammation via anti-inflammatory cytokines ad chemokines production. Among pro-inflammatory cytokine suppressed by the p62-plasmid are IL-1, IL-6, TNF, while anti-inflammatory cytokines IL-4, IL-10 and IL-1RA were increased (15, 32). .

Although the mechanism of the p62-plasmid action in OA is out the scope of this study, we believe that anti-inflammatory effect may play an important role. Further studies of the p62-plasmid are warranted with larger number of canine patients and a control group. In these studies measurements of inflammation biomarkers might support a better understanding of mechanism of the p62-plasmid. Also, the present study justifies analysis of potential applications of the p62-plasmid as anti-pain medicine.

## Figures and Tables

**Table 1:** Patient characteristics

#	Sex	Age	Weight	Breed
1	f	11,5	35	Doberman
2	m	4,5	34	Akita
3	m	8,5	6	Mixed
4	m	6,5	50	Bernernese Mountain Dog
5	f	13,5	49	South Russian shepherd dog
6	f	13	6	Mixed
7	f	11	35	Labrador retriever
8	f	8	35	Labrador retriever
9	m	5	3	Pomeranian Spitz
10	f	9,5	35	Labrador retriever
11	f	11,5	32	German Shepherd
12	f	11	35	Labrador retriever
13	m	14	40	Labrador retriever
14	m	12	40	Labrador retriever
15	m	6	3,2	Yorkshire Terrier
16	f	6	9,5	Beagle
17	f	7,5	7,5	Fox Terrier

Medium (range) 9 (4–14) 28(3–50)

**Table 2:** Efficacy of p62 plasmid treatment in dogs with OA

Dog id	Time point	PSS	PIS	QoL
1	Pre	6.00	7.00	Fair (1)
	Post	0.25	0.17	Good (2)
2	Pre	8.00	7.50	Good (2)
	Post	3.75	4.00	Good (2)
3	Pre	4.25	8.33	Fair (1)
	Post	0.00	0.00	Excellent (3)
4	Pre	3.75	1.33	Fair (1)
	Post	1.00	1.00	Good (2)
5	Pre	6.25	7.83	Fair (1)
	Post	5.25	5.83	Fair (1)
6	Pre	4.25	7.00	Fair (1)
	Post	3.25	3.17	Fair (1)
7	Pre	5.00	4.83	Fair (1)
	Post	1.50	2.00	Good (2)
8	Pre	1.50	4.50	Fair (1)
	Post	0.00	0.00	Excellent (3)
9	Pre	5.00	7.17	Fair (1)
	Post	2.00	7.00	Poor (0)
10	Pre	6.00	7.33	Poor (0)
	Post	3.25	4.67	Good (2)
11	Pre	4.00	6.17	Fair (1)
	Post	2.00	2.33	Good (2)
12	Pre	7.50	8.67	Fair (1)
	Post	8.00	7.33	Fair (1)
13	Pre	6.00	6.67	Fair (1)
	Post	6.25	7.33	Fair (1)
14	Pre	9.00	9.50	Fair (1)
	Post	4.50	4.17	Good (2)
15	Pre	5.25	0.50	Fair (1)
	Post	0.75	0.00	Excellent (3)
16	Pre	8.00	5.50	Fair (1)
	Post	3.50	3.17	Good (2)
17	Pre	5.25	5.67	Fair (1)
	Post	4.25	4.50	Good (2)

**Table 3:** Change in QoL of dogs after the treatment with p62 plasmid, n (%)

QoL,	Before	After
Poor (0)	1 (5.9%)	1 (5.9%)
Fair (1)	15 (88%)	4 (24%)
Good (2)	1 (5.9%)	9 (53%)
Excellent (3)	0 (0%)	3 (18%)

QoL = p < 0.001
